# Dissecting the Association of Apolipoprotein E Gene Polymorphisms With Type 2 Diabetes Mellitus and Coronary Artery Disease

**DOI:** 10.3389/fendo.2022.838547

**Published:** 2022-02-08

**Authors:** Lei Wu, Yan Zhang, Hong Zhao, Guodong Rong, Peijun Huang, Fang Wang, Ting Xu

**Affiliations:** ^1^ Department of Laboratory Medicine, The First Affiliated Hospital of Nanjing Medical University, Nanjing, China; ^2^ National Key Clinical Department of Laboratory Medicine, Nanjing, China

**Keywords:** apolipoprotein E, polymorphism, type 2 diabetes mellitus, coronary artery disease, meta-analysis

## Abstract

**Background:**

Apolipoprotein E (*APOE*) gene mediates lipoprotein clearance and is one of the most studied candidate genes for type 2 diabetes mellitus (T2DM) and coronary artery disease (CAD). This study was performed to determine the association between *APOE* polymorphisms and T2DM with and without CAD, and its effect on plasma lipid levels in a Chinese population.

**Methods:**

A total of 1,414 subjects involving 869 patients and 545 health individuals were recruited. These patients were categorized into three distinct groups: 264 in T2DM group, 401 in CAD group, and 204 in T2DM+CAD group. Logistic regression analysis was used to obtain odds ratio (OR) and 95% confidence interval (CI) in predicting the risk probability of *APOE*. Besides, a meta-analysis was preformed to integrate an evaluation index to evaluate their associations.

**Results:**

Genotype frequency ratio of genotype ϵ3/4 and allele ϵ4 among the CAD patients with or without T2DM was obviously increased. Compared with ϵ3/3 genotype, the ϵ3/4 genotype had a significant increased risk of CAD (adjusted OR = 1.90, 95% CI = 1.30–2.77) and T2DM+CAD (adjusted OR = 1.95, 95% CI = 1.24–3.08). In the meta-analysis, four studies were included and provided a strong evidence for the *APOE* ϵ4 mutation elevating the risk of CAD in patients with T2DM (ϵ3/ϵ4+ϵ4/ϵ4 *vs*. ϵ3/ϵ3, OR = 1.51, 95% CI = 1.13–2.02). In the T2DM group, the plasma levels of low-density lipoprotein cholesterol (LDL-C) showed significant difference among the three APOE isoforms. The high-density lipoprotein cholesterol (HDL-C) levels of CAD patients with ϵ4-bearing genotypes were lower than those with ϵ3/3 genotype.

**Conclusions:**

Our results indicate that *APOE* gene polymorphisms are related to CAD with or without T2DM and have influence on lipid profiles in both T2DM and CAD patients.

## Introduction

Type 2 diabetes mellitus (T2DM) is a common chronic metabolic disease characterized by high levels of sugar in the blood and is prevalent throughout the world. The incidence of T2DM is rising at an alarming rate attributed to changing dietary patterns, increasing life expectancy, and westernization of lifestyles in developing countries (1). T2DM frequently coexists with various complications such as hypertension and dyslipidemia and is also known as a major independent risk factor for coronary artery disease (CAD) (2). Cardiovascular disease including CAD is increased in T2DM subjects, which is associated with significant morbidity and mortality. Patients with T2DM have two- to fourfold greater risk of developing CAD compared to individuals without diabetes (3). The development of CAD in the setting of T2DM due to a complex combination of various risk factors plays important role in the beginning and the evolution of atherosclerosis (4). The inherited aspect of risk factors is most often a number of genes interacting with each other or with the environmental factors. Therefore, managing genetic risk factors for T2DM and CAD may improve the understanding of these disease and result in better clinical management.

**Figure 1:**
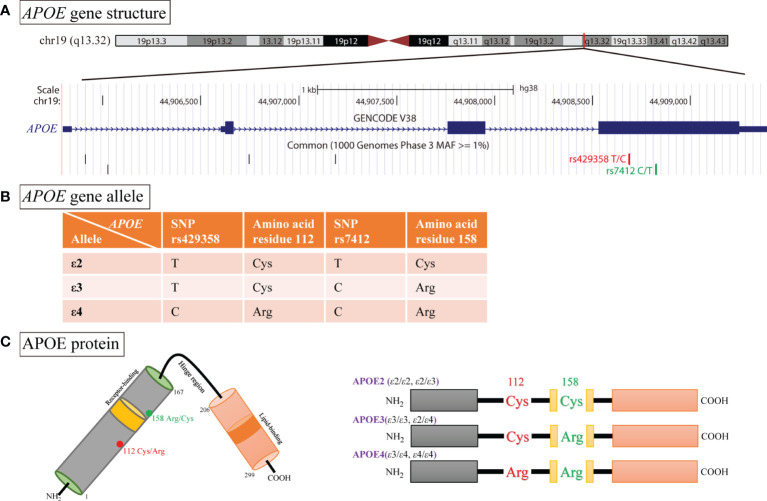
Schematic illustration of *APOE* genotype and APOE isoform. **(A)** Functional single nucleotide polymorphisms (SNPs) in the *APOE* gene viewed in UCSC genome browser. **(B)** Two SNPs (rs429358 and rs7412) in exon 4 of *APOE* generate three major allelic variants (ϵ2, ϵ3, and ϵ4). **(C)** APOE protein is a polypeptide chain with 299 amino, and the APOE2, APOE3, and APOE4 isoforms differ from one another at amino acid residues 112 or 158.

Apolipoprotein E (*APOE*) gene maps in the long arm of chromosome 19 at position q13.32, which encodes a multifunction glycoprotein containing 299 amino acids (**Figure 1A**). It acts as cholesterol carrier and is involved in mediating the transportation and metabolism of lipids (5). As shown in **Figure 1B**, two single-nucleotide polymorphisms (SNPs) in *APOE*, namely, rs429358 (T>C) and rs7412 (C>T), gives rise to three major alleles: ϵ2 (rs429358-T, rs7412-T), ϵ3 (rs429358-T, rs7412-C), and ϵ4 (rs429358-C, rs7412-C). Therefrom, the three alleles yield six different genotypes, of which three are homozygous, namely, ϵ2/ϵ2, ϵ3/ϵ3, and ϵ4/ϵ4, and three are heterozygous, namely, ϵ2/ϵ3, ϵ2/ϵ4, and ϵ3/ϵ4. Besides, these variants encode three different protein isoforms: APOE2 (ϵ2/ϵ2, ϵ2/ϵ3; Cys112/Cys158), APOE3 (ϵ2/ϵ4, ϵ3/ϵ3; Cys112/Arg158), and APOE4 (ϵ3/ϵ4, 4/ϵ4; Arg112/Arg158, **Figure 1C**) (6). Previous studies have shown that variants of APOE could govern the metabolism of lipoproteins. Allele ϵ4 is associated with lower plasma high-density lipoprotein cholesterol (HDL-C) level but had higher plasma levels of low-density lipoprotein cholesterol (LDL-C), total cholesterol (TC), and triglycerides (TG), when compared with ϵ3 allele. Meanwhile, the presence of ϵ2 is usually coupled with lower plasma levels of LDL (7, 8). Referring to those previous studies, evidence suggests that a functional interaction between *APOE* polymorphisms and LDL receptor (LDL-R) influences the risk of CAD and T2DM, and ϵ4 allele has higher affinity to LDL-R than other alleles (9, 10). It is thus likely that the effects of APOE ϵ4 are due to overproduction of LDL or fewer LDL-R, overwhelming the limited ability of mediating the clearance of lipoproteins (10, 11). In contrast, Larifla et al. has reported that the lack relationship between *APOE* polymorphisms and CAD in Afro-Caribbean people (12). Besides, according to a recent meta-analysis including 13 eligible studies, *APOE* gene ϵ4 allele had a significant increased risk for CAD patients with T2DM, whereas the ϵ2 variation had null association (13). Consequently, we conducted a case–control study to investigate the association of *APOE* polymorphisms with T2DM and CAD in a Chinese population and its potential role in lipid metabolism.

## Methods

### Study Population

The studied subjects were recruited from The First Affiliated Hospital of Nanjing Medical University (Nanjing, China) from January 2018 to December 2019. Our study was approved by Ethical Committee of The First Affiliated Hospital of Nanjing Medical University, and all donors signed a written informed consent before enrollment. Only Chinese subjects aged 18 years or above were recruited. Questionnaires were used to collect the information of age, sex, genetic family history, medical history, and lifestyle habits. Other clinical and biochemical data such as blood pressure, dyslipidemia, and blood glucose were obtained from clinical and laboratory examinations. Dyslipidemic or hyperlipidemic feature matches the following conditions: TC > 5.17mmol/L (200 mg/dl), TG > 1.69 mmol/L (150 mg/dl), LDL-C > 3.38 mmol/L (130 mg/dl), or HDL-C < 1.03 mmol/L (40 mg/dl). A fasting plasma glucose (FPG) level ≥7.0 mmol/L (126 mg/dl) or a 2-h plasma glucose ≥11.1 mmol/L (200 mg/dl) meets the threshold for the diagnosis of diabetes.

According to the above criteria, studied subjects were classified into four groups. First is the T2DM group that included 264 subjects that fulfilled the diabetes diagnostic criteria of FPG ≥7.0 mmol/L or were under treatment with oral antidiabetic drugs. Second is the CAD group that consisted of 401 subjects with at least 50% stenosis in a major coronary artery or one of their branches defined by coronary angiography. Third is the T2DM+CAD group included 204 subjects diagnosed to have diabetes complicated with coronary artery disease. Exclusion criteria included type 1 diabetes mellitus, malignant tumors, liver and kidney diseases, metabolic disorders, and autoimmune diseases. Fourth is the control group that included 545 healthy individuals without hyperlipidemia, hypertension, cardiovascular diseases, and diabetes.

### *APOE* Genotyping

Genomic DNA was isolated from leukocytes of the peripheral blood using a commercial kit following the manufacturer’s protocols (Sinochip, Zhuhai, China). DNA concentration and purity were estimated by 260 and 280 nm (optical density, OD) light absorbance on a Nanodrop 2000™ spectrophotometer (Thermo Fisher Scientific, Waltham, MA, USA). Two *APOE* SNPs (rs429358 and rs7412) were genotyped using a detection kit (GeneChip Assay, Sinochip, Zhuhai, China). All samples were amplified according to the manufacturer’s instructions, and then, the amplified products were assayed by the fully automated GeneChip detection system (Sinochip, Zhuhai, China).

### Lipid Profiles

After an overnight fast of at least 12 h, venous blood samples were collected from these patients. Plasma TC, TG, HDL-C, LDL-C, and FPG were quantified using the Beckman biochemical assembly line.

### Systematic Review and Meta-analysis

We conducted a literature search for all studies that evaluated the association of *APOE* polymorphisms with T2DM and CAD in the PubMed database up to December 2021. The following key terms were used in the search: “apolipoprotein E” or “*APOE*,” “polymorphism,” “CAD,” and “T2DM.” References cited in each retrieved article were also manually scanned to discover additional eligible studies. Articles were recruited for this meta-analysis if they fitted the following criteria: (1) investigated the associations between *APOE* polymorphisms and CAD in patients with T2DM, (2) sufficient data for estimating the odds ratios (ORs) and 95% confidence intervals (CIs), and (3) published in English. Exclusion criteria were (1) duplication of previous data and (2) not using coronary angiography to confirm CAD.

### Statistical Analysis

Continuous data such age and lipid profile were compared using Student’s *t*-test or Wilcoxon test for two groups and Kruskal–Wallis test for more than two groups. Categorical variables (sex and *APOE* genotypes) were expressed as frequency and compared using Pearson’s χ^2^ test or Fisher’s exact test. Hardy–Weinberg equilibrium was conducted to evaluate the allele and genotype difference among groups. The associations between *APOE* polymorphism and diseases were estimated by computing crude or adjusted ORs and 95% CIs from unconditional logistic regression. All the statistical analyses were done with R 4.0.1, and two-sided p-value <0.05 was considered statistically significant.

## Results

### Characteristics and Clinical Features of Subjects

The demographic and clinical features of 1,414 included individuals are summarized in **Table 1**. The data from normal controls were used as a reference to compare with the data obtained from three observation groups consisted of patients with T2DM, CAD, and T2DM+CAD. A significant difference in age was found between control group (mean age, 67.2 years) and the observation groups (T2DM, 69.5 years; CAD, 65.3 years; T2DM+CAD, 70.5 years), implying a higher risk of developing T2DM with increasing age. Gender was equally distributed in CAD group with 111 female patients and 290 male patients and in T2DM+CAD group with 65 female and 139 male patients compared to control group with 206 female and 339 male subjects. There was also significant sex difference between CAD patients and the controls (p = 0.001) due to the high prevalence of male patients among CAD compared to control group (72.3% vs. 62.2%). Besides, T2DM and T2DM+CAD groups had significantly higher levels of TG than the normal control group. Patients with CAD or T2DM had lower levels of HDL-C than the controls. Therefore, the TG/HDL-C ratio was calculated, and this index was significantly higher among the observation groups.

**Table 1:** Clinical characteristics and genotype distribution of APOE gene in different groups.

Variables		Control	T2DM		CAD		T2DM+CAD	
		n = 545	n = 264	p	n = 401	p	n = 204	p
**Age** (years)		67.2 ± 13.4	69.5 ± 12.7	**0.022**	65.3 ± 12.3	**0.029**	70.5 ± 13.0	**0.003**
**Sex**								
Female		206 (37.8%)	85 (32.2%)	0.120	111 (27.7%)	**0.001**	65 (31.9%)	0.132
Male		339 (62.2%)	179 (67.8%)		290 (72.3%)		139 (68.1%)	
**Lipid profile** (mmol/L)								
TC		4.25 ± 0.96	4.30 ± 1.23	0.974	4.33 ± 1.13	0.635	4.29 ± 1.34	0.385
TG		1.42 ± 0.91	1.72 ± 1.30	**<0.001**	1.49 ± 0.89	0.238	1.86 ± 1.45	**<0.001**
HDL-C		1.15 ± 0.33	1.05 ± 0.28	**<0.001**	1.09 ± 0.26	**0.032**	1.02 ± 0.27	**<0.001**
LDL-C		2.58 ± 0.71	2.65 ± 0.89	0.506	2.69 ± 0.86	0.173	2.70 ± 1.03	0.794
**TG/HDL-C ratio**		1.38 ± 1.12	1.87 ± 1.88	**<0.001**	1.49 ± 1.10	**0.032**	2.08 ± 2.13	**<0.001**
***APOE* genotypes**								
APOE2	ϵ2/ϵ2	6 (1.10%)	3 (1.14%)	1.000	1 (0.25%)	0.249	0 (0%)	**–**
	ϵ2/ϵ3	87 (15.96%)	47 (17.80%)	0.509	52 (12.97%)	0.198	27 (13.24%)	0.355
APOE3	ϵ2/ϵ4	6 (1.10%)	2 (0.76%)	1.000	3 (0.75%)	0.741	4 (1.96%)	0.473
	ϵ3/ϵ3	387 (71.01%)	171 (64.77%)	0.072	268 (66.83%)	0.169	132 (64.71%)	0.096
APOE4	ϵ3/ϵ4	58 (10.64%)	39 (14.77%)	0.090	75 (18.70%)	**<0.001**	39 (19.12%)	**0.002**
	ϵ4/ϵ4	1 (0.18%)	2 (0.76%)	1.000	2 (0.50%)	0.577	2 (0.98%)	0.182
***APOE* alleles**								
ϵ2		105 (9.63%)	55 (10.42%)	0.621	57 (7.11%)	0.052	31 (7.60%)	0.222
ϵ3		919 (84.31%)	428 (81.06%)	0.101	663 (82.67%)	0.340	330 (80.88%)	0.112
ϵ4		66 (6.06%)	45 (8.52%)	0.066	82 (10.22%)	**<0.001**	47 (11.52%)	**<0.001**

Data are presented as mean ± SD, or numbers (N) and percentage. p-value: comparison between T2DM/CVD/T2DM+CAD group and control group. Groups were compared using Student’s t-test or Wilcox test (for continuous variables) and Pearson’s χ^2^ test or Fisher’s exact test (for categorical variables). Bold values denote statistical significance at the p < 0.05 level.

### *APOE* Genotype and Allele Frequencies of Subjects

Our extracted genomic DNA was of good quality with an OD260/OD280 ratio between 1.7 and 2.0. The genotype distributions of all included groups were in Hardy–Weinberg equilibrium (p > 0.05), exhibiting group representation. The most frequent genotype was ϵ3/ϵ3 in our subjects, followed by ϵ3/4 and ϵ2/3 genotypes, while ϵ2/2, ϵ2/4, and ϵ4/4 genotypes were the lowest, indicating that there was a significant difference in the distribution frequency of genotype ϵ3/4 among these groups. Compared with the control group, the genotype ratio of ϵ3/4 obviously increased in the CAD and T2DM+CAD groups (p < 0.001 and p = 0.002, **Table 1**). The differences in ϵ4 allele frequency distribution between the CAD and T2DM+CAD groups and the control group were considered statistically significant (both p < 0.001).

### Association of *APOE* Polymorphism With Diseases

**Figure 2:**
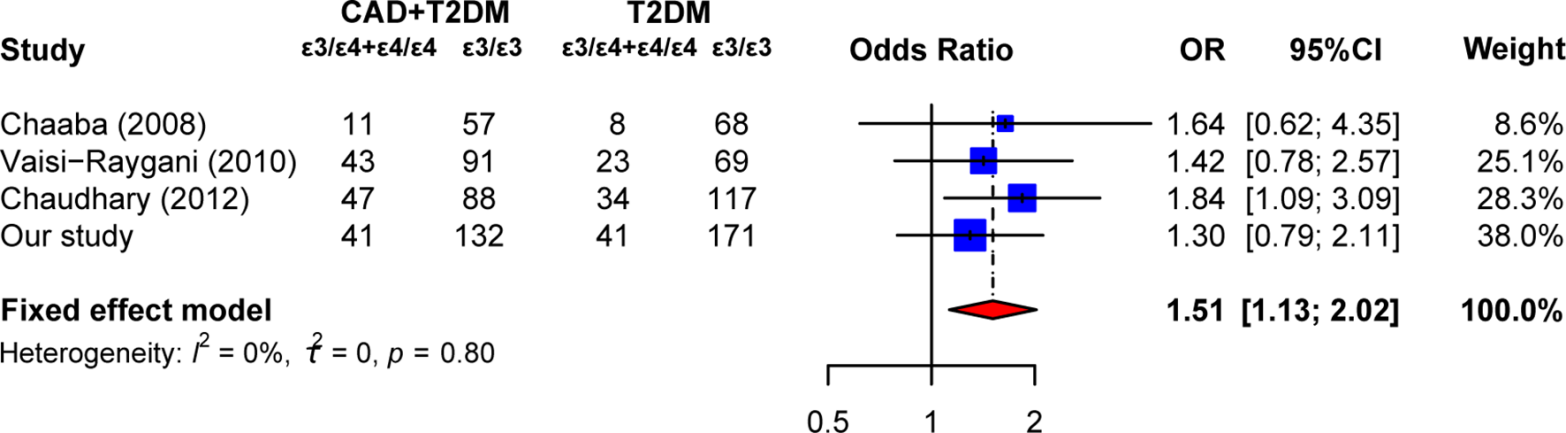
Summary estimates of the association between the *APOE* ϵ4 mutation (ϵ3/ϵ4+ϵ4/ϵ4 vs. ϵ3/ϵ3) and the risk of coronary artery diseases in type 2 diabetes patients. Each study is displayed by a square whose center represents the odds ratio (OR), the area of the square is proportional to the weight of studies, and the horizontal line indicates the 95% confidence interval (CI).

Logistic regression was performed to evaluated the correlation between *APOE* polymorphism and T2DM or CAD. As shown in **Table 2**, when the ϵ3/3 genotype was used as the reference, the ϵ3/4 genotype had a significant increased risk of CAD and T2DM+CAD (adjusted OR = 1.90, 95% CI = 1.30–2.77 for CAD; adjusted OR = 1.95, 95% CI = 1.24–3.08 for T2DM+CAD). Furthermore, *APOE* allele ϵ4 appeared to increase the risk of developing CAD without or with T2DM, with adjusted OR of 1.72 (95% CI, 1.22–2.42) and 1.97 (95% CI, 1.32–2.93), respectively, compared with the allele ϵ3. However, allele ϵ4 was not found to be associated with the risk of CAD in T2DM (ϵ4 *vs.* ϵ3, OR = 1.36, 95% CI = 0.88–2.09). These results encouraged us to conduct a meta-analysis to explore the association between *APOE* allele ϵ4 and T2DM complicated with CAD. According to the above search criteria, three articles (14–16) and our study were included in the meta-analysis (**Supplementary Figure S1**). **Figure 2** displays that ϵ3/4 + ϵ4/4 genotype increased the risk of developing CAD in T2DM patients, with a pooled OR of 1.51 (95% CI, 1.13–2.02), compared to the ϵ3/3 genotype. Besides, no heterogeneity was detected, indicating that the pooled results of this meta-analysis were statistically steady and robust.

**Table 2:** Comparison of APOE genotypes and alleles frequency between the control group and the case group.

Variables	Control	T2DM				CAD				T2DM+CAD			
APOE genotypes	n	n	OR (95% CI)	OR (95% CI)^a^	p^a^	n	OR (95% CI)	OR (95% CI)^a^	p^a^	n	OR (95% CI)	OR (95% CI)^a^	p^a^
ϵ2/ϵ2	6	3	1.13 (0.28-4.58)	1.19 (0.29-4.83)	0.813	1	0.24 (0.03-2.01)	0.22 (0.03-1.82)	0.158	0	–	–	–
ϵ2/ϵ3	87	47	1.22 (0.82-1.82)	1.22 (0.82-1.82)	0.334	52	0.86 (0.59-1.26)	0.86 (0.59-1.25)	0.427	27	0.91 (0.57-1.46)	0.90 (0.56-1.46)	0.673
ϵ2/ϵ4	6	2	0.75 (0.15-3.78)	0.77 (0.15-3.86)	0.748	3	0.72 (0.18-2.91)	0.69 (0.17-2.81)	0.605	4	1.96 (0.54-7.03)	1.84 (0.5-6.72)	0.357
ϵ3/ϵ3	387	171	Reference	Reference	–	268	Reference	Reference	–	132	Reference	Reference	–
ϵ3/ϵ4	58	39	1.52 (0.98-2.37)	1.45 (0.93-2.27)	0.102	75	**1.87 (1.28-2.72)**	**1.90 (1.30-2.77)**	**0.001**	39	**1.97 (1.26-3.1)**	**1.95 (1.24-3.08)**	**0.004**
ϵ4/ϵ4	1	2	4.53 (0.41-50.25)	5.18 (0.46-57.94)	0.182	2	2.89 (0.26-32.01)	2.23 (0.20-24.85)	0.515	2	5.86 (0.53-65.19)	6.27 (0.56-70.17)	0.136
**Alleles**													
ϵ2	105	55	1.13 (0.80-1.59)	1.13 (0.80-1.60)	0.484	57	0.75 (0.54-1.06)	0.74 (0.53-1.04)	0.081	31	0.82 (0.54-1.25)	0.82 (0.53-1.25)	0.346
ϵ3	919	428	Reference	Reference	–	663	Reference	Reference	–	330	Reference	Reference	–
ϵ4	66	45	1.46 (0.99-2.18)	1.42 (0.96-2.12)	0.082	82	**1.72 (1.23-2.42)**	**1.72 (1.22-2.42)**	**0.002**	47	**1.98 (1.34-2.94)**	**1.97 (1.32-2.93)**	**< 0.001**

a Adjusted for age and sex. Bold values denote statistical significance at the p < 0.05 level.

### Relationship Between APOE Polymorphism and Lipid Profiles

**Figure 3:**
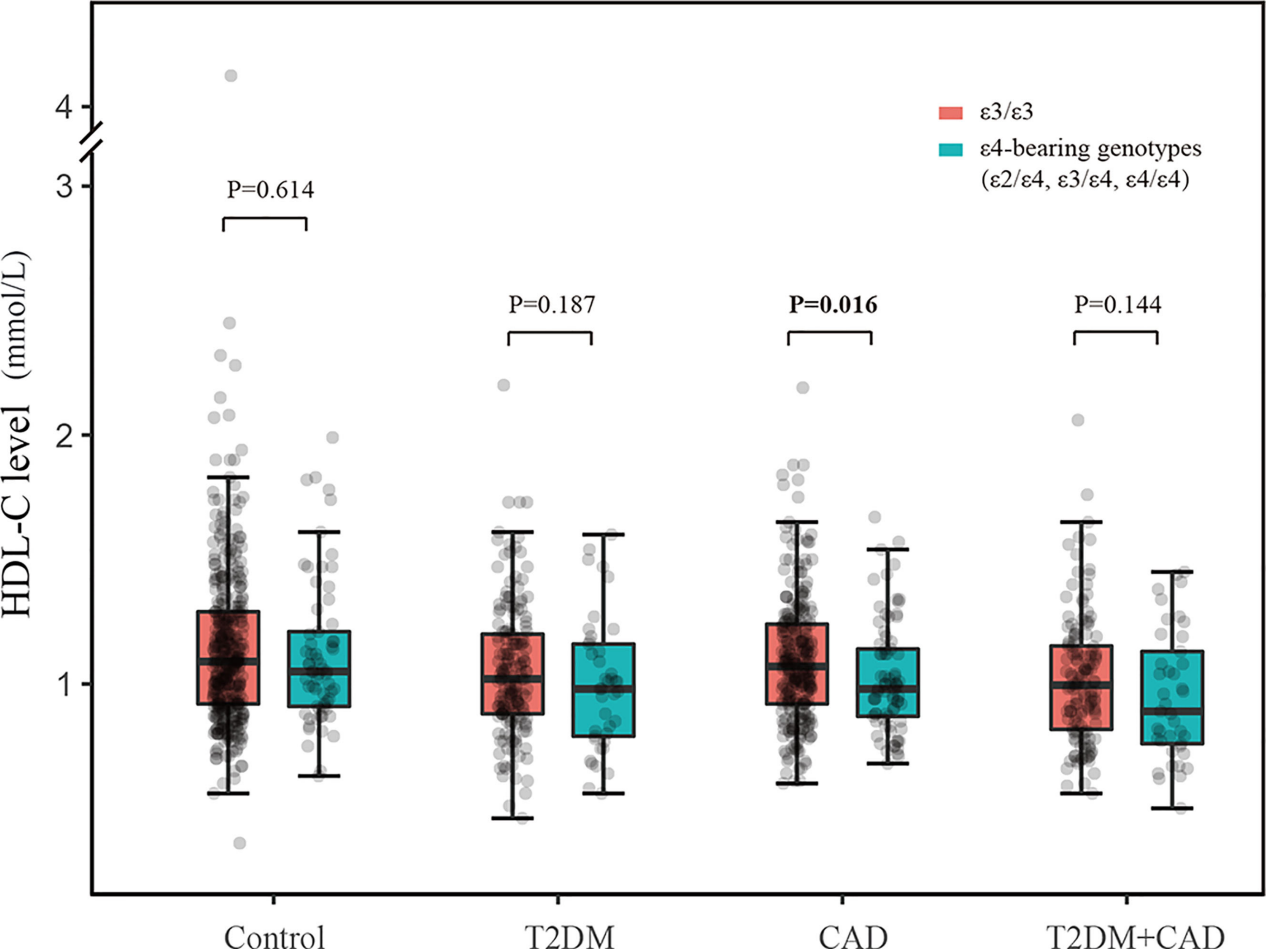
Correlation between plasma HDL-C level values and *APOE* ϵ4-bearing genotypes (ϵ2/ϵ4, ϵ3/ϵ4, and ϵ4/ϵ4).

We analyzed the blood lipid profiles in subjects with different APOE genotypes. In the control and T2DM group, the levels of LDL-C showed significant difference among APOE2, APOE3, and APOE4 individuals; in the CAD group, APOE4 patients had lower levels of HDL-C than APOE3 and APOE2 patients. However, there was no significant difference in TG/HDL-C ratio across different APOE genotype groups (**Supplementary Table S1**). The frequencies of the ϵ4 allele might contribute to the difference in APOE distribution between observation groups and control group. Therefore, we analyzed the correlation between ϵ4-bearing genotypes (ϵ2/ϵ4, ϵ3/ϵ4, and ϵ4/ϵ4) and plasma HDL-C expression. As shown in **Figure 3**, ϵ3/ϵ3 genotype as a reference, ϵ4-bearing genotypes had significant decreased levels of HDL-C in CAD group (p = 0.016) but not in the other three groups. We also examined the association between ϵ4-bearing genotypes and TG/HDL-C ratio, and yet, the difference was not statistically significant (**Supplementary Figure S2**).

## Discussion

T2DM, a chronic condition disease, induced by a genetic predisposition together with environmental factors, is a well-established risk factor for CAD. T2DM and its related cardiovascular complications propose specific challenges at diverse stages of the life. *APOE* polymorphisms have been reported to significantly associate with risk for T2DM and CAD, which were considered as the most influential genetic risk factors. Here, we carried out several experiments to evaluate the association between the *APOE* ϵ2/ϵ3/ϵ4 polymorphisms with the risk of T2DM and CAD. When combined with the analysis the polymorphism of *APOE* and blood lipid levels, these results provided new understanding on the correlation between *APOE* gene and T2DM patients with CAD.

Our study provided evidence for the significant correlation between *APOE* ϵ3/ϵ4 genotype and an elevated risk of CAD without or with T2DM. After adjusting for age and sex, logistic regression analysis showed that ϵ4 allele increased the risk of CAD by 1.72 times, compared with ϵ3 allele. Besides, T2DM patients carrying ϵ4 allele had 1.97-fold higher risk of CAD as compared to the controls. Our data indicated that ϵ4 allele was an independent risk factor for CAD but not for T2DM. Previous studies have investigated the probable associations between *APOE* polymorphisms and patients with T2DM or CAD. Chaudhary et al. reported that the ϵ4 allele was significantly higher in both T2DM and CAD as compared with controls (16). The independent predictor of individuals carrying ϵ4 allele remained significantly associated with both CAD (adjusted OR = 2.32, 95% CI = 1.17–4.61) and T2DM (adjusted OR = 2.04, 95% CI = 1.07–3.86). El−Lebedy et al. found that the frequencies of ϵ3/ϵ4 genotype and ϵ4 allele were increased in both T2DM patients and cardiovascular disease (CVD) patients as compared with controls but were significant only in CVD patients (17). Diabetic patients who carried ϵ3/ϵ4 genotype had 2.4-fold increased risk of developing CVD (95% CI, 1.14–5.19), and the ϵ4 allele was associated with 2.23-fold higher CVD risk (95% CI, 1.09–4.59). A recent meta-analysis including 13 studies provided evidence that there were significant associations between ϵ4 allele and the risk of CAD in patients with T2DM (ϵ3/ϵ4 vs. ϵ3/ϵ3, OR = 1.69, 95% CI = 1.38–2.08; ϵ4/ϵ4 vs. ϵ3/ϵ3, OR = 2.72, 95% CI = 1.61–4.60) (13). Combined with our own data and setting of strict inclusion and exclusion criteria, our meta-analysis found that ϵ4 mutation could elevate the risk of CAD in patients with T2DM. Therefore, we may conclude that ϵ3/4 genotypes and ϵ4 allele of *APOE* contributed to CAD in T2DM patients.

One outcome from this work is the levels of HDL-C in CAD patients with ϵ4-bearing genotypes (ϵ2/ϵ4, ϵ3/ϵ4, and ϵ4/ϵ4) were lower than patients with ϵ3/ϵ3 genotype. It was well-known that the HDL-C levels were important factors of blood lipid levels. The relationship between ϵ4 allele and lipid profile remained controversial. Li et al. found that the *APOE* ϵ4 carrier had a lower HDL-C than the ϵ2 allele but not for ϵ3 allele in a Chinese population of CAD (18). In a recent study on Kashmiri population, the CAD patients carrying ϵ4 allele had significantly lower HDL-C levels (19). Chaaba et al. found that the ϵ4 allele was only associated with elevated LDL-C concentration and with CAD in type 2 diabetic men in Tunisian population, showing that gender interacted with the effects of *APOE* polymorphism (14). Therefore, these inconsistent findings might be complicated by considerable differences in the allele frequency distributions among different ethnic populations.

Current evidence showed that APOE is a versatile glycoprotein that plays a central role in lipoprotein metabolism (20). Although the three APOE isoforms differ in only one or two amino acids, these slight changes affect the structure and alter the affinity to lipoproteins. APOE3 binds preferentially to HDL, whereas APOE4 shows an enhanced lipid bind ability of VLDL particles, which impairs their lipolytic processing in the circulation, resulting in a more pro-atherogenic lipoprotein–cholesterol distribution. Therefore, abnormalities of lipoprotein metabolism may partly explain that the plasma HDL-C levels were lowest in APOE4 patients compared to APOE3 and APOE2 patients in CAD group. Besides, excessive amounts of circulant lipids may affect systemic inflammation and insulin resistance (IR) (21, 22). Previous studies reported the TG/HDL-C ratio as predictor of IR (22). A higher ratio indicated a large amount of circulant lipids. Our study showed that the T2DM+CAD group had the highest TG/HDL-C ratio than the control, T2DM, and CAD groups. Then, we assessed whether the APOE isoforms were associated with IR, but no association was observed. Confirming results of previous studies, the relationship between *APOE* allele frequencies and IR was controversial (23–25).

There are several limitations that should be pointed out as follows. First, this case–control study was hospital based, and the selection bias was inevitable. Therefore, we adjusted for potential confounding factors such as age and sex to minimize the bias in logistic regression. Second, adult populations showed specific mutations not only owing to genetic background. Life habits, diets, climate, pollution, and even pandemic might influence it. As this study recruited subjects aged 18 years or above, subgroup analysis for age (children and adults) could not be performed. Meanwhile, we did not have the detailed information of some risk factors (smoking, diet, and physical activity) in the development of CAD and T2DM, and the gene–environment interactions analysis was not conducted. Third, there was no significant association between *APOE* alleles and T2DM in our study, which was not consistent with previous study (26, 27), and the results from our single-center study required external validation in other training programs. A final comment on the low number of individuals in some subgroups (control, T2DM, CAD, and T2DM+CAD for ϵ2/ϵ2, ϵ2/ϵ4 and ϵ4/ϵ4) should be taken up with care and caution.

In conclusion, although the *APOE* ϵ4 allele was not found to be associated with T2DM, it increased the risk of CAD and related to the development of T2DM with CAD. Different APOE isoforms were also linked to variations in lipid and lipoprotein levels in circulation.

## Data Availability Statement

The original contributions presented in the study are included in the article/**Supplementary Material**. Further inquiries can be directed to the corresponding author.

## Ethics Statement

The studies involving human participants were reviewed and approved by the Ethical Committee of The First Affiliated Hospital of Nanjing Medical University. The patients/participants provided their written informed consent to participate in this study.

## Author Contributions

TX and LW designed the research. LW, YZ, HZ, GR, PH, FW, and TX conducted research. LW and YZ analyzed data. LW wrote the initial draft of the manuscript. FW and TX revised the manuscript. All authors contributed to the article and approved the submitted version.

## Conflict of Interest

The authors declare that the research was conducted in the absence of any commercial or financial relationships that could be construed as a potential conflict of interest.

## Publisher’s Note

All claims expressed in this article are solely those of the authors and do not necessarily represent those of their affiliated organizations, or those of the publisher, the editors and the reviewers. Any product that may be evaluated in this article, or claim that may be made by its manufacturer, is not guaranteed or endorsed by the publisher.
