# Supplementary material for: Dissecting the Association of Apolipoprotein E Gene Polymorphisms With Type 2 Diabetes Mellitus and Coronary Artery Disease
Source: Front Endocrinol (Lausanne). 2022 Feb 8;13:838547. doi: 10.3389/fendo.2022.838547 (PMC8861372; doi:10.3389/fendo.2022.838547)
Supplement: Supplementary file 1 [file DataSheet_1.docx]

Supplementary Table 1: Relationship between APOE polymorphism and lipid profiles in different groups

| **Variables** |  | **TC** | **P** |  | **TG** | **P** |  | **HDL-C** | **P** |  | **LDL-C** | **P** |  | **TG/HDL-C ratio** | **P** |
| --- | --- | --- | --- | --- | --- | --- | --- | --- | --- | --- | --- | --- | --- | --- | --- |
| **Control** |  |  |  |  |  |  |  |  |  |  |  |  |  |  |  |
| APOE2 | ε2/ε2 | 4.11±0.97 | 0.204 |  | 1.57±0.97 | 0.200 |  | 1.18±0.28 | 0.153 |  | 2.43±0.70 | **0.013** |  | 1.46±1.10 | 0.581 |
|  | ε2/ε3 |  |  |  |  |  |  |  |  |  |  |  |  |  |  |
| APOE3 | ε2/ε4 | 4.25±0.92 |  |  | 1.39±0.94 |  |  | 1.14±0.34 |  |  | 2.58±0.69 |  |  | 1.37±1.18 |  |
|  | ε3/ε3 |  |  |  |  |  |  |  |  |  |  |  |  |  |  |
| APOE4 | ε3/ε4 | 4.46±1.13 |  |  | 1.34±0.63 |  |  | 1.11±0.31 |  |  | 2.83±0.83 |  |  | 1.29±0.69 |  |
|  | ε4/ε4 |  |  |  |  |  |  |  |  |  |  |  |  |  |  |
| **T2DM** |  |  |  |  |  |  |  |  |  |  |  |  |  |  |  |
| APOE2 | ε2/ε2 | 4.02±1.22 | 0.057 |  | 1.89±1.01 | 0.127 |  | 1.05±0.33 | 0.418 |  | 2.41±0.91 | **0.031** |  | 2.15±2.02 | 0.158 |
|  | ε2/ε3 |  |  |  |  |  |  |  |  |  |  |  |  |  |  |
| APOE3 | ε2/ε4 | 4.43±1.25 |  |  | 1.69±1.39 |  |  | 1.06±0.26 |  |  | 2.75±0.89 |  |  | 1.80±1.85 |  |
|  | ε3/ε3 |  |  |  |  |  |  |  |  |  |  |  |  |  |  |
| APOE4 | ε3/ε4 | 4.06±1.12 |  |  | 1.61±1.26 |  |  | 1.00±0.27 |  |  | 2.51±0.81 |  |  | 1.85±1.82 |  |
|  | ε4/ε4 |  |  |  |  |  |  |  |  |  |  |  |  |  |  |
| **CAD** |  |  |  |  |  |  |  |  |  |  |  |  |  |  |  |
| APOE2 | ε2/ε2 | 4.31±1.07 | 0.777 |  | 1.49±0.84 | 0.938 |  | 1.17±0.32 | **0.011** |  | 2.59±0.78 | 0.946 |  | 1.42±1.13 | 0.555 |
|  | ε2/ε3 |  |  |  |  |  |  |  |  |  |  |  |  |  |  |
| APOE3 | ε2/ε4 | 4.36±1.16 |  |  | 1.48±0.91 |  |  | 1.10±0.26 |  |  | 2.71±0.89 |  |  | 1.48±1.08 |  |
|  | ε3/ε3 |  |  |  |  |  |  |  |  |  |  |  |  |  |  |
| APOE4 | ε3/ε4 | 4.24±1.03 |  |  | 1.51±0.87 |  |  | 1.02±0.22 |  |  | 2.67±0.81 |  |  | 1.62±1.17 |  |
|  | ε4/ε4 |  |  |  |  |  |  |  |  |  |  |  |  |  |  |
| **T2DM+CAD** |  |  |  |  |  |  |  |  |  |  |  |  |  |  |  |
| APOE2 | ε2/ε2 | 4.37±1.78 | 0.292 |  | 2.01±1.38 | 0.661 |  | 1.11±0.33 | 0.079 |  | 2.65±1.26 | 0.135 |  | 1.95±1.45 | 0.589 |
|  | ε2/ε3 |  |  |  |  |  |  |  |  |  |  |  |  |  |  |
| APOE3 | ε2/ε4 | 4.37±1.33 |  |  | 1.79±1.44 |  |  | 1.02±0.26 |  |  | 2.78±1.04 |  |  | 2.01±2.14 |  |
|  | ε3/ε3 |  |  |  |  |  |  |  |  |  |  |  |  |  |  |
| APOE4 | ε3/ε4 | 3.98±0.98 |  |  | 2.00±1.57 |  |  | 0.95±0.26 |  |  | 2.44±0.77 |  |  | 2.46±2.43 |  |
|  | ε4/ε4 |  |  |  |  |  |  |  |  |  |  |  |  |  |  |

Data are presented as mean ± SD

Groups were compared using Kruskal-Wallis test.

Supplementary Figure 1
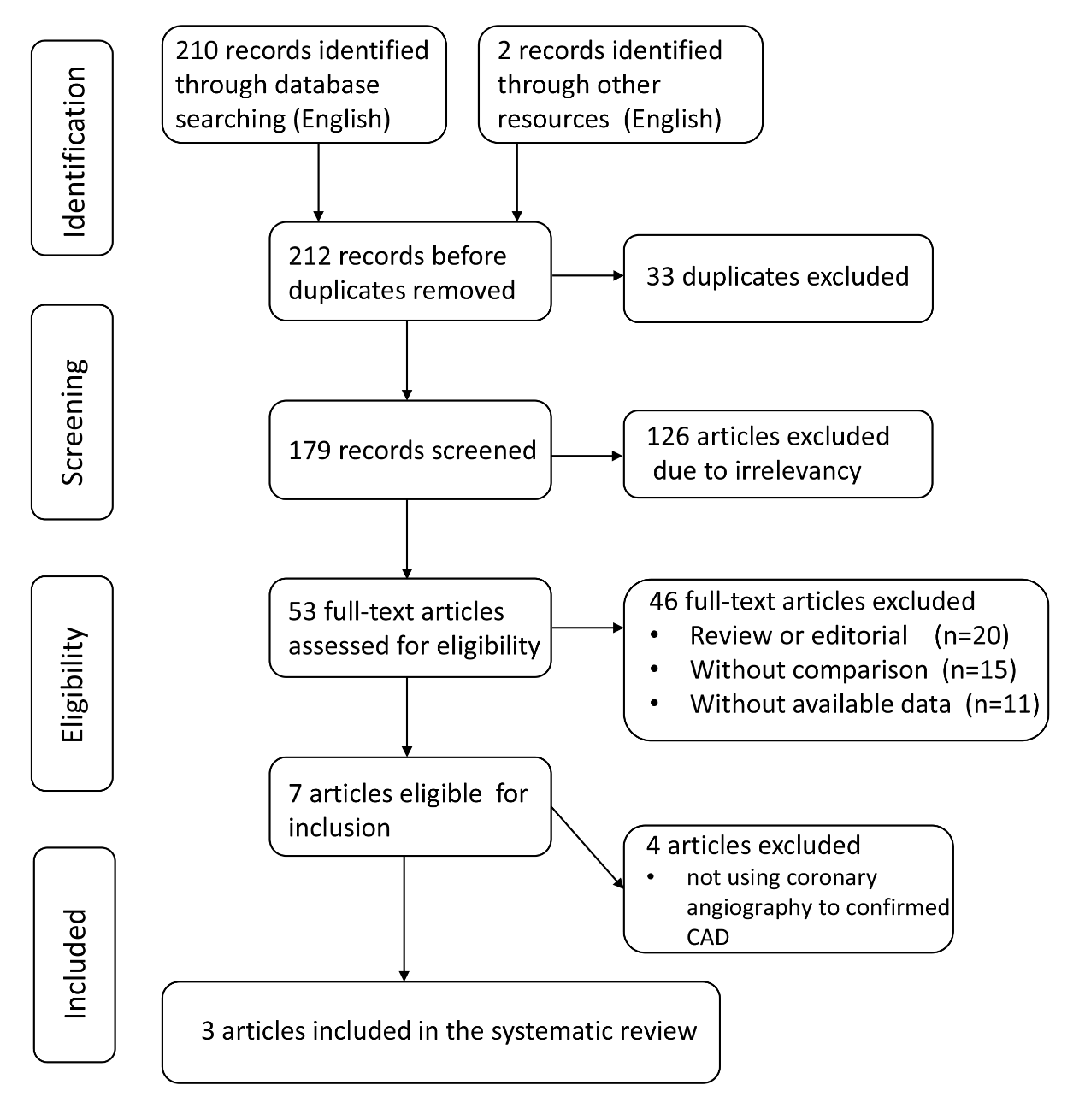


Flowchart of articles identified with criteria for inclusion and exclusion.

Supplementary Figure 2


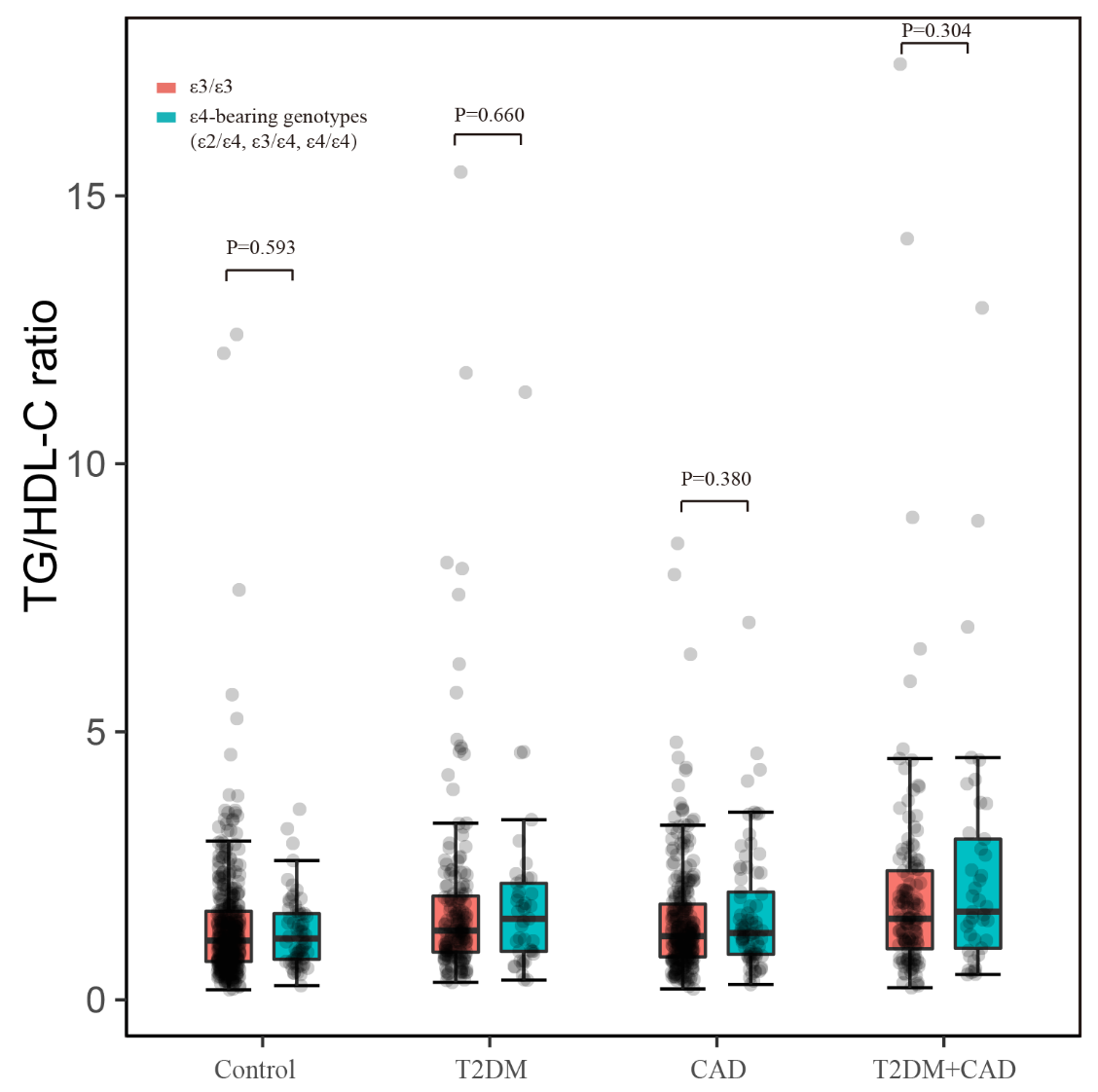


Correlation between TG/HDL-C ratio and *APOE* ε4-bearing genotypes (ε2/ε4, ε3/ε4, ε4/ε4).
